# Effect and Safety of Mycophenolate Mofetil in Idiopathic Pulmonary Fibrosis

**DOI:** 10.1155/2011/849035

**Published:** 2011-11-01

**Authors:** Argyris Tzouvelekis, Evangelos Bouros, Anastasia Oikonomou, Paschalis Ntolios, George Zacharis, George Kolios, Demosthenes Bouros

**Affiliations:** ^1^Department of Pneumonology, University Hospital of Alexandroupolis, Democritus University of Thrace, 68100 Alexandroupolis, Greece; ^2^Laboratory of Pharmacology, Democritus University of Thrace, 68100 Alexandroupolis, Greece; ^3^Department of Radiology, University Hospital of Alexandroupolis, Democritus University of Thrace, 68100 Alexandroupolis, Greece

## Abstract

*Background*. Idiopathic pulmonary fibrosis (IPF) is a progressive fibrotic interstitial lung disease with ineffective treatment. Mycophenolate mofetil (MMF) is an immunomodulatory agent which inhibits lymphocyte proliferation. *Objective*. We sought to determine the safety and efficacy profile of MMF in IPF patients. *Methods*. We retrospectively identified ten patients, who met the ATS/ERS 2000 criteria for IPF and received MMF 2 gr/day for 12 months. All of them had routine laboratory, pulmonary function and radiological (high resolution computed tomography-HRCT) data available and were enrolled in the study. Forced vital capacity (FVC), total lung capacity (TLC), diffusion capacity of the lung for carbon monoxide (DL_CO_), 6-minute walking distance (6MWD), HRCT scans and routine laboratory data at treatment onset were compared with respective values 12 months after treatment onset. *Results*. There were no significant alterations in FVC, TLC, DL_CO_ and 6MWD pre- and 6 and 12 months post-treatment. HRCT evaluation showed deterioration of the total extent of disease (*P* = 0.002) and extent of ground-glass opacity (*P* = 0.02). No cases of clinically significant infection, leucopenia, or elevated liver enzymes were recorded. *Conclusions*. MMF is a safe therapeutic modality which failed to show a beneficial effect both in functional and radiological parameters in a small cohort of IPF patients.

## 1. Introduction

Idiopathic pulmonary fibrosis (IPF) is an irreversible, devastating, progressive type of lung fibrosis that culminates in a fatal outcome irrespective of treatment [1]. Despite innumerable research studies and rapid expansion of scientific knowledge, IPF pathogenesis still remains elusive and controversial [2–5]. Recent data strongly suggest that the mechanisms driving IPF reflect abnormal deregulated wound healing in response to multiple sites of ongoing alveolar epithelial injury of unknown origin leading to fibroblast activation and exaggerated accumulation of extracellular matrix into the lung parenchyma [2–6]. Therefore, our present understanding of the molecular and cellular pathways has resulted in the testing of therapeutic approaches that modulate specific inflammatory and fibrotic mediators. With a gradually increasing worldwide incidence and no proven therapies other than lung transplantations, IPF treatment is a major challenge for chest physicians [7–9]. 

Mycophenolate mofetil (MMF), an inhibitor of lymphocytes proliferation through blockade of inosine monophosphate dehydrogenase and interference with purine biosynthesis, is commonly used to prevent rejection following solid-organ transplantation [10–14]. Its clinical utility has been expanded for the treatment of several autoimmune and renal disorders [15]. MMF languished in relative obscurity until the past 5 years when it emerged to function not only as an anti-inflammatory but also as an antiproliferative agent by downregulating the expression of several critical growth factors including transforming growth factor- (TGF-) *β*. This property makes it an attractive candidate drug for the treatment of fibrotic lung disease [16]. 

However, there is a serious lack of knowledge and clinical experience regarding its safety, tolerability, and efficacy in patients with IPF, a disease with ineffective treatment and a dismal prognosis. This retrospective study seeks to determine the safety profile and demonstrate the effectiveness of MMF treatment during the disease course in a small cohort of IPF patients. 

## 2. Patients and Methods

### 2.1. Patients

This is a retrospective, single-center trial estimating the safety and efficacy of MMF for IPF treatment. After approval by the Local Ethics Committee and the Institutional Scientific Review Board (reference number 45/4 Scientific Committee-16/11/2009) patients (*n* = 10) were retrospectively identified who met the ATS/ERS 2000 criteria for IPF [1] and received, on an off-label basis, MMF 2 gr/day for >6 months, between September 2006 and October 2008. Mean time from diagnosis drug initiation was 9 ± 2 months. Patients who had no serial routine laboratory, functional, and radiological data available were excluded from the analysis (*n* = 0). Patients were evaluated on an outpatient basis at the Department of Pneumonology, University Hospital of Alexandroupolis, Democritus University of Thrace, Greece. All patients gave informed consent. 

### 2.2. Assessment of High-Resolution Computer Tomography (HRCT) Data

High-resolution CT sections (1 mm) were acquired supine, at full inspiration, at 10 mm intervals reconstructed with bone algorithm using a spiral CT scanner (GE Prospeed Series). The scans were scored by a thoracic radiologist with 9 years of experience (A. Oikonomou), blinded to clinical and lung function information [17]. HRCT images were scored at five predetermined levels: (1) origin of great vessels, (2) main carina, (3) pulmonary venous confluence, (4) halfway between the third and fifth section, and (5) immediately above the right hemidiaphragm. HRCT variables evaluated were total disease extent, the extent of reticular pattern, the extent of ground-glass, the proportion of ground-glass opacity, and the coarseness of reticular disease.

#### 2.2.1. Extent of Disease

The total extent of interstitial lung disease was estimated to the nearest five percent in each of the five sections, with global extent of disease on HRCT computed as the mean of the scores. 

#### 2.2.2. Extents of Individual Patterns

HRCT patterns were subdivided into reticular disease (innumerable interlacing line shadows that were fine, intermediate, or coarse, with variable associated distortion of the lung architecture) and ground-glass attenuation (a hazy increase in lung parenchymal attenuation, with preservation of bronchial and vascular markings) [18]. The relative proportions of the two patterns, estimated in each section, were multiplied by the total extent of disease to provide separate extent scores for each pattern, with the global scores computed as mean values, as for overall disease extent. From these scores, the contribution made by ground glass to overall disease extent was calculated (proportion of ground glass). 

#### 2.2.3. Coarseness of Reticulation

The most severe disease in each section was quantified as grade 0 = ground glass attenuation alone, grade 1 = fine intralobular fibrosis, grade 2 = microcystic honeycombing (air spaces less than or equal to 4 mm in diameter), and grade 3 = macrocystic honeycombing (air spaces greater than 4 mm in diameter). The total coarseness score was the summed score for all five levels (range 0 to 15).

### 2.3. Statistical Analysis

Continuous data are presented as medians with ranges or mean + SD. The paired two-tailed Student's *t*-test was used to assess statistically significant differences in functional parameters at baseline and 12 months after treatment. Linear regression analysis was used to determine whether there was any improvement in FVC, TLC, and DL_CO_ 6 and 12 months after MMF treatment initiation. The paired Wilcoxon signed ranks test, nonparametric tests were employed to analyse radiological findings. Statistical analysis was performed with SPSS software, version 17.0. 

## 3. Results

### 3.1. Baseline Characteristics

Baseline characteristics of patients enrolled in the study are shown in Table 1. As demonstrated, all patients were male, 9 out of 10 (90%) were ex-smokers, at the time of treatment initiation. Six out of 10 patients (60%) had histopathological biopsy proven IPF/usual interstitial pneumonia (UIP) whereas in the remaining four diagnosis was based on the radiological UIP pattern. Seven out of 10 patients (70%) were previously untreated whereas the remaining three patients had used low doses of corticosteroids (two under 20 mgrs and one under 10 mgrs of methylprednisdone daily), at the time of treatment initiation. In addition, three patients (30%) had pulmonary hypertension at the time of MMF initiation (sPAP greater than 60 mmHg, with an overall mean sPAP = 37.2 + 19.6 mmHg) estimated by echocardiography and were started with endothelin-receptor antagonists (one with 250 mgrs of bosentan and the remaining two with 10 mgrs of ambrisentan). Underlying autoimmunity was excluded by the absence of signs of arthritis, morning stiffness, sclerodactyly, photosensitivity, and Raynaud's phenomenon coupled with negative immunologic profile (antinuclear antibodies-ANA, anti-ds DNA antibodies, and rheumatoid factor) in eight out of ten patients. Two patients had positive ANA antibodies, with a negative remaining immunologic profile and physical examination, in the remaining two patients, which could not verify the presence of an autoimmune disorder.

### 3.2. MMF Treatment Failed to Show Disease Improvement Based on Pulmonary Function Parameters 

**Figure 1:**
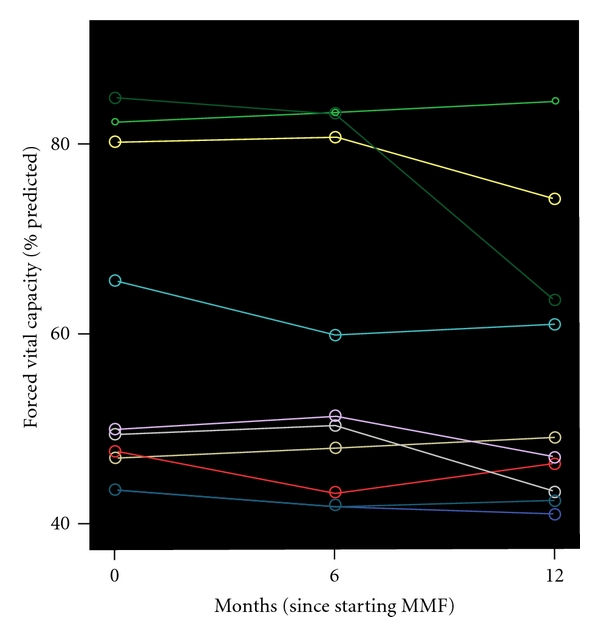
Forced vital capacity (FVC) fluctuations over time for each subject. Each line represents measurements made in a single subject. A time point 0 month indicates when MMF treatment was commenced.

**Figure 2:**
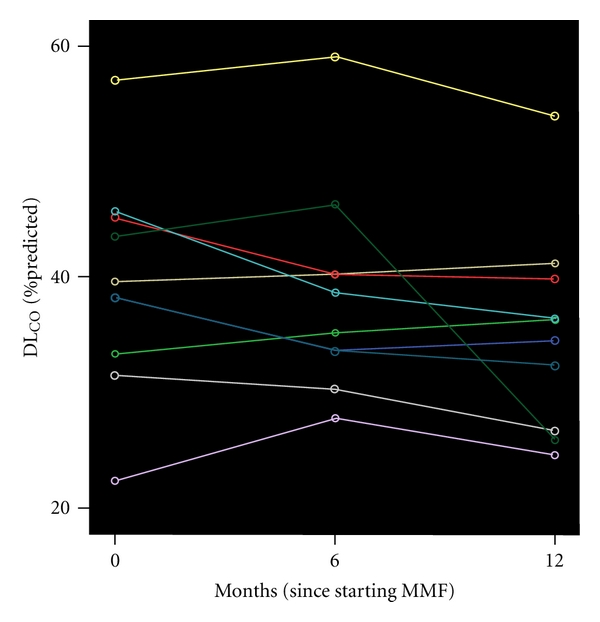
Diffusion capacity of the lung for carbon monoxide (DL_CO_) fluctuations over time for each subject. Each line represents measurements made in a single subject. A time point 0 month indicates when MMF treatment was commenced.

As demonstrated in Table 2 and Figures 1 and 2, MMF treatment failed to show a beneficial effect as assessed by pulmonary function parameters. Linear regression analysis showed that FVC (*P* = 0.228, *P* = 0.081), TLC (*P* = 0.70, *P* = 0.081), and DL_CO_ (*P* = 0.47, *P* = 0.053) did not change significantly both 6 and 12 months after MMF treatment initiation, respectively. In addition, MMF administration was associated in 6-minute walking distance (6MWD) at baseline and 12 month after treatment (*P* = 0.09). Finally, no alterations in alveolar-arterial gradient of oxygen tension (P_A-a_O_2_) between pre- and 12 posttreatment levels (*P* = 0.67) were noted. 

### 3.3. MMF Treatment Was Associated with Disease Progression Based on High-Resolution Computed Tomography (HRCT) Data

Eight out of 10 IPF patients treated with MMF had HRCT evaluation before and after treatment with mean time interval between the two HRCT scans of 12 months. The remaining 2 patients had HRCT evaluation only before initiation of MMF treatment because they died due to acute exacerbation and therefore there was no data available.

Among the eight patients who had HRCT evaluation both before and after initiation of MMF treatment the mean HRCT scores for the HRCT variables are shown in Table 3. Statistical analysis showed that there was disease progression based on the total extent of disease (*P* = 0.002) and extent of ground-glass opacity (*P* = 0.02) while there was no significant change concerning the extent of reticular pattern, the proportion of ground-glass opacity, and the coarseness of reticular disease (*P* > 0.05). 

### 3.4. Clinical and Laboratory Acceptable Safety Profile

Patients were followed for 12 months with routine laboratory tests, including liver enzymes and white blood cells count. No cases of liver toxicity, clinically significant infection, and leucopenia were recorded during MMF treatment. In addition, MMF was well tolerated by all patients with no development of abdominal pain, nausea, or vomiting episodes that could lead to treatment discontinuation or dosage reduction. The above data suggest that MMF has an acceptable safety and tolerability profile. 

## 4. Discussion

This is the first report in the literature investigating the safety and efficacy profile of a novel immunomodulatory agent, MMF, given to a small cohort of IPF patients. We retrospectively collected laboratory, functional, and radiological data and demonstrated a readily acceptable safety profile with no important adverse events justifying drug discontinuation or dosage reduction. Regarding drug effectiveness, MMF treatment failed to show a beneficial effect as assessed by functional parameters (FVC, TLC, DL_CO_, 6MWD, and P_A-a_O_2_) while disease progression based on HRCT data, as assessed by using a highly standardized scoring system, was seen. 

The pharmacological treatment that is currently available for IPF is clearly inadequate [8, 19–25]. The emergence of novel and powerful tools have provided scientists and physicians with numerous avenues of investigation with clinical applications to greatly improve our understanding of IPF pathogenesis. However, this fatal disease still remains without proven therapies other than lung transplantations given to a small minority of individuals [7, 9]. In view of the current disappointing survival data arising from large prospective placebo-controlled clinical trials, many chest physicians worldwide apply other therapeutic regimens to attempt IPF treatment. 

MMF has been extensively used to downregulate host-immune response following solid-organ transplantation and therefore to prevent rejection [10–14, 26]. In addition, MMF has been also proven effective in the treatment of several autoimmune and renal disorders, including systemic lupus erythematosus [15]. Based on the versatile anti-inflammatory and immunomodulatory properties of its active metabolite, mycophenolic acid, MMF treatment has been recently applied with promising results in patients with systemic sclerosis (SSc) with interstitial lung involvement. 

In particular, Liossis et al. demonstrated a beneficial effect of MMF both in functional and radiological parameters in five patients with SSc-associated alveolitis [27]. Moreover, MMF administration was well tolerated and safe showing no serious adverse events. Further extending their results, Gerbino et al., retrospectively identified 13 patients with SSc-interstitial lung disease who were treated with MMF and suggested that MMF improves vital capacity 12 months after treatment [28]. Findings were also replicated by another group of investigators in a small cohort of SSc patients with interstitial lung disease, where authors reported a beneficial effect of MMF on the functional status of these patients [29]. Since T cells seem to play a vital role in the pathogenesis of scleroderma and mycophenolic acid inhibits, via blockage of inosine monophosphate, T-cell proliferation and downregulates their intracellular adhesion to endothelial cells, it is highly possible that a beneficial effect of this drug might be anticipated. 

Fueled by this prospect and based on the aforementioned promising results, US investigators have recently launched a large multicentre randomized clinical trial to compare the beneficial effect in lung function parameters of a 2-year course of MMF with those of a 1-year course of oral cyclophosphamide, in patients with symptomatic scleroderma-related interstitial lung disease. This trial is still ongoing and its results are greatly anticipated (for more information go to  http://clinicaltrials.gov/). 

In past years, the role of T cells in the pathogenesis of IPF was relatively overlooked mainly due to the disappointing results of corticosteroid treatment. However, interest in the role of autoimmunity in IPF pathophysiology was revived by a study showing that CD4+ cells in IPF patients are in a highly activated status and proliferate rigorously when stimulated with IPF lung extracts, suggesting the presence of an autoimmune process through recognition of self-antigens [30]. In line with this premise, our study group demonstrated a numerical and functional impairment of regulatory T cells (Tregs), a specific subset of T cells which is essential for the control of immunologic tolerance and the prevention of autoimmunity, in IPF patients [31]. Furthermore, this global defect was highly correlated with indicators of disease severity, such as functional parameters, implicating an involvement of Tregs in the fibrotic process. 

Despite relative enthusiasm arising from the above findings implicating autoimmunity in the pathogenesis of IPF and highlighting novel therapeutic targets with clinical applications, functional and radiological results from our current study would downplay the role of T cells during disease progression. It is therefore conceivable to speculate that the inability of the drug to be proven efficacious lies both in the previously suggested minor contribution of T cells in the pathogenesis of IPF [32] as well as in the inevitable progressive clinical course. 

Nevertheless it is important to clarify that there might be a minority of IPF patients that would benefit from immunosuppressive agents such as MMF, including those waiting for lung transplantation as it happens with patients waiting for renal transplants where MMF is used to prevent solid-organ rejection. Based on MMF's immunosuppressive and antiproliferative properties and since MMF is often part of the posttransplant immunosuppressive regimen in these patients MMF might be considered for use before subjecting the patient to major surgery [33]. Larger prospective studies in highly selective group of IPF patients are needed to extract efficacy outcomes. 

Our study has a number of limitations. First of all, it is retrospective in its nature and underpowered. Secondly, based on our data it is unknown whether stabilization of functional parameters could be attributed to therapeutic intervention or simply represents a bystander of disease clinical course. Alternatively, it is impossible to establish a clear relationship between drug effect and disease outcome mainly due to study design. Larger, prospective randomized studies are needed to extract outcomes of scientific rigidity and verify our results, as occurred with scleroderma associated interstitial lung involvement. Finally, it is important to underline that in our case series all the functional parameters showed a gradual decline, even though statistically insignificant, evidence that may be attributed to lack of study power. 

Collectively, MMF was well tolerated and safe, showing no clinically significant side effects while it failed to show a beneficial effect in disease progression as assessed by functional and radiological parameters. Our main findings underline the current disappointing status in the treatment field of this debilitating disease and highlight the necessity for future large, prospective, randomised clinical trials of novel therapeutic agents with versatile properties targeting multiple pathogenetic pathways. 

## Figures and Tables

**Table 1:** Baseline characteristics of the study population.

Characteristics	Baseline data
Subjects	10
Male	10
Female	0
Age (years)	63 (44–73)
Smokers	0
Ex-smokers	10
Nonsmokers	0
Prior treatment (steroids) received	3
Other treatment received	3
VATS	6
FVC %pred	59.2 ± 17.1
TLC %pred	53.9 ±10.2
DL_CO_ %pred	39.4 ± 9.3
6MWD (meters)	441 ±124.5
P_A-a_O_2_ (mmHg)	27.4 ±11.5
sPAP (by echocardiography) mmHg	37.2 ± 19.6

Data are presented as median (range), no (total) or mean ± SD, unless stated otherwise.

6MWD: 6-minute walking distance, FVC: forced vital capacity, NA: nonapplicable, P_A-a_O_2_: alveolar-arterial gradient of oxygen tension, sPAP: systolic pulmonary artery pressure, TLC: total lung capacity.

**Table 2:** FVC, TLC, DL_CO_, 6MWD, and P_A-a_O_2_ at baseline and 6 and 12 month after MMF treatment.

	Baseline	6 months	12 months	*P*-value^1^	*P*-value^2^
FVC (%) pred	59.2 ± 17.1	58.2 ± 17.2	55 ± 14.9	0.228	0.081
TLC (%)	53.9 +10.2	53.6 +12.3	52 ±12.8	0.702	0.081
DL_CO _ (%)	39.4 + 9.3	38.5 + 9	35.2 + 8.8	0.47	0.053
6MWD	441 +124	NA	421 + 123	NA	0.09
P_A-a_O_2_	27.4 +11.5	NA	27.7+11.2	NA	0.67

Data are presented as mean ± SD unless stated otherwise, *P*-value^1^: between baseline and 6 months, *P*-value^2^: between baseline and 12 months;

6MWD: 6-minute walking distance, FVC: forced vital capacity, NA: nonapplicable, P_A-a_O_2_: alveolar-arterial gradient of oxygen tension, TLC: total lung capacity.

**Table 3:** HRCT scores before and after MMF treatment.

	Disease extent 0 month	Reticular extent 0 month	GGO extent 0 month	Coarseness reticulation 0 month	Proportion GGO 0 month	Disease extent 12 months	Reticular extent 12 months	GGO extent 12 months	Coarseness reticulation 12 months	Proportion GGO 12 months
1	23	23	5,2	9	22,6	28	18,7	9,3	9	33,21
2	23	10,5	12,5	8	54	52	34,8	17,2	10	32
3	25	22,5	2,5	11	10	28	17,2	10,8	11	38,57
4	59	31,3	27,7	12	47	67	35,1	31,9	12	47,6
5	38	18,2	19,8	10	52,1	64	40,4	23,6	13	36,8
6	32	19,3	15,3	9	36,9	33	29,9	19,2	11	36,9
7	33	20,1	14,9	11	37,8	35	31,2	25,2	13	35,8
8	31	22,1	11,2	10	36,9	46	30,9	26,2	10	39,2
Mean	33	20,7	13,6	10	37,02	44	29,7	20,42	11	37,5
*P*-value						0.002*	>0.05	0.02*	>0.05	>0.05

HRCT: high-resolution computed tomography, MMF: mycophenolate mofetil, GGO: ground-glass opacity.

## Abbreviations

6MWD: 6-minute walking distance
DL_CO_: Diffusion capacity of the lung for carbon monoxide
FVC: Forced vital capacity
GGO: Ground-glass opacity
HRCT: High-resolution computed tomography
IPF: Idiopathic pulmonary fibrosis
MMF: Mycophenolate mofetil
NA: Non applicable
P_A-a_O_2:_: Alveolar-arterial gradient of oxygen tension
sPAP: Systolic pulmonary artery pressure
SSc: Systemic sclerosis
TGF: Transforming growth factor
TLC: Total lung capacity
Tregs: regulatory T cells
UIP: Usual interstitial pneumonia.
